# A Gelatin-Based Film with Acerola Pulp: Production, Characterization, and Application in the Stability of Meat Products

**DOI:** 10.3390/polym17131882

**Published:** 2025-07-06

**Authors:** Vitor Augusto dos Santos Garcia, Giovana de Menezes Rodrigues, Victória Munhoz Monteiro, Rosemary Aparecida de Carvalho, Camila da Silva, Cristiana Maria Pedroso Yoshida, Silvia Maria Martelli, José Ignacio Velasco, Farayde Matta Fakhouri

**Affiliations:** 1 Faculty of Agricultural Sciences, São Paulo State University (UNESP), Av. Universitária, 3780, Botucatu 18610-034, SP, Brazil; 2 Faculty of Engineering, Federal University of Grande Dourados, Dourados 79804-970, MS, Brazil; giovanamr@usp.br (G.d.M.R.); vicmunhozm@gmail.com (V.M.M.); silviamartelli@ufgd.edu.br (S.M.M.); 3 Faculty of Animal Science and Food Engineering, University of São Paulo, Av. Duque de Caxias Norte, 225, Pirassununga 13635-900, SP, Brazil; rosecarvalho@usp.br; 4 Technology Department, State University of Maringá, Av. Ângelo Moreira da Fonseca 1800, Umuarama 87506-370, PR, Brazil; camiladasilva.eq@gmail.com; 5 Institute of Environmental, Chemical and Pharmaceutical Science, UNIFESP—Federal São Paulo University, Rua São Nicolau 210, Diadema 09913-030, SP, Brazil; cristiana.yoshida@unifesp.br; 6 Poly2 Group, Department of Materials Science and Engineering, Universitat Politècnica de Catalunya (UPC BarcelonaTech), ESEIAAT, Carrer de Colom, 11, 08222 Terrassa, CT, Spain

**Keywords:** antioxidant compounds, active packaging, food preservation

## Abstract

The objective of this work was to produce and characterize active gelatin–acerola packaging films based on gelatin incorporated with different concentrations of acerola pulp and applied to evaluate the stability of meat products in packaging. The active films were produced by casting using gelatin (5%), sorbitol (0,1%), and acerola pulp (60, 70, 80, and 90%). The characterization of the acerola pulp was carried out. Visual aspects, thickness, pH, water vapor permeability, and total phenolic compounds were characterized in the films. The commercial acerola pulp presented the characteristics within the identity and quality standards. A good film formation capacity was obtained in all formulations, presenting the color parameters tending to red coloration, characteristic of the acerola pulp. The total phenolic compounds content ranged from 2.88 ± 70.24 to 3.94 ± 96.05 mg GAE/100 g, with 90 g of acerola pulp per 100 g of filmogenic solution. This film formulation was selected to apply in a vacuum pack of beef and chicken samples, analyzing the weight loss, color parameters, pH, water holding capacity, shear strength after 9 days of refrigeration storage, and soil biodegradability. Additionally, beef and chicken (in nature) were stored under the same conditions without using the wrapping film. The beef and chicken samples showed greater water retention capacity and color maintenance over the storage period compared to the control (without film addition). This way, active gelatin–acerola films can be considered a sustainable packaging alternative to preserve meat products.

## 1. Introduction

Lipid oxidation in raw meat during storage can affect its quality and acceptability, mainly due to its shelf life [1]. Antioxidants from different sources can control lipid oxidation. Muscle tissue is mainly susceptible to oxidation due to high concentrations of unsaturated lipids, heme pigments, catalysts, and other types of oxidative agents [2]. Antioxidants are usually added to some types of meat products. In general, antioxidants are synthetic compounds that can cause several problems for human health related to their toxic potential and carcinogenicity [3]. Alternatives such as active packaging materials with different functionalities are being studied for applications in meat products. According to Han et al. [4], the development of quality indicator films for food products, particularly to detect changes in food quality, is an emerging trend in food packaging. To evaluate the quality of salmon, the authors developed a film made of whey protein nanofibers, enriched with anthocyanin and glycerol, which demonstrated color changes in response to pH, from dark pink to gray, correlated with the deterioration of salmon. This advancement can significantly contribute to food safety [4]. Arrowroot starch films with grape pomace extract have the potential to become pH-sensitive films for use in monitoring the freshness of protein-rich foods as well as for use as bioactive packaging, The effect of this combination was demonstrated when used on tilapia fish fillets. The authors concluded that color changes were observed for GPE solutions and film-forming solutions containing GPE: pink for acidic pH, gray for neutral pH, and green to yellow for basic pH. The film with 40% GPE transitioned from red to green during the fish deterioration process, highlighting its functionality as a visual sensor of meat freshness [5].

Natural polymer materials have been used for packaging meat products [3,6,7,8], mainly to increase shelf life, maintaining organoleptic characteristics and reducing lipid oxidation. Among the natural polymers, gelatin is widely applied in food products. It is obtained by the thermal denaturation of animal collagen, has excellent biodegradability, and has good film-forming properties [9]. Due to the recent search for packaging that is not harmful to the environment, gelatin has also been widely used as a packaging material and coating material for food products. For active edible films based on green tea extract and gelatin for the coating of fresh sausage, authors found that the use of active gelatin film (1.0% of green tea extract) kept the TBARS indexes of fresh sausage samples lower than the standard (without coating) and of films containing only gelatin, after 48 days of storage [10].

When studied as packaging for chicken breast, this protein reduced the loss of mass in the fillets when compared to the control chicken. In addition, the authors used tea tree oil in some formulations, which allowed the packaging to have an antibacterial effect against *Salmonella* sp. [11]. In addition, the authors emphasized that films with added tea tree oil can be effectively used as a natural antimicrobial and antioxidant agent in food packaging applications. A potential material based on betulin/hydroxypropyl-beta-cyclodextrin inclusion complex nanofibers (BE/HPβCD-IC-NF) was used as packaging material and was shown to have antioxidant and antimicrobial properties. In addition, it offers significant biodegradability and is environmentally friendly, making it possible to extend shelf life while remaining ecologically safe [12].

One of the alternatives to producing active packaging is based on compounds obtained from natural sources. There are four main methods for incorporating bioactive compounds into biopolymeric films [13]: using biopolymers that are naturally bioactive, derived from agricultural products or byproducts; mixing bioactive compounds directly with biopolymers during film production; encapsulating bioactive compounds into micro- or nanoparticles, and then mixing them physically with the biopolymer during film formation; and encapsulating the compounds, but instead of mixing, spraying the micro- or nanoparticles onto the biopolymer during film production.

In this type of packaging, substances are released or absorbed from the packaging material to the product, which maintains the quality and sensory properties of the food product during storage and distribution [14]. An active and biodegradable package with freshness detection properties for meat and fish was developed based on cellulose nanofibers and cellulose acetate, incorporating anthocyanins extracted from *Melastoma malabathricum* seeds to activate the film for the detection of freshness ammonia through color change [15]. Active packaging material based on different blends of nisin, EDTA, poly (butylene adipate terephthalate), and thermoplastic starch blends were obtained by extrusion, verifying that films containing EDTA and nisin inhibited lipid degradation and microbial growth in pork meat [16].

Acerola (*Malpighia emarginata*) has an average production of 29.65 tons per hectare per year, which can be continuous, depending on production conditions [17]. Several studies have been conducted due to their high potential for antioxidant capacity application [18,19,20,21]. The addition of acerola extract as a natural antioxidant extended the shelf life of hamburgers, improving the color product and delaying lipid oxidation, reducing the rancid flavor [22]. Edible films obtained from acerola puree with alginate, corn syrup, and cellulose fibers were characterized by mechanical and barrier properties [23]. Previous studies have explored the antioxidant potential of acerola when associated with the incorporation of polysaccharides. However, the application of biodegradable gelatin packaging material incorporating natural active compounds, such as acerola puree, in the stability of food storage is still incipient. In this context, this study presents an innovative approach by developing biodegradable active films based on gelatin incorporated with acerola puree, aimed at preserving meat products. The use of whole fruit, rich in natural antioxidant compounds, represents a sustainable and functional alternative, promoting the valorization of agro-industrial by-products. Its application in vacuum-packed meat highlights the potential of these films as eco-friendly substitutes for conventional packaging in the food industry.

The present work aimed to produce and characterize active gelatin-based films by incorporating acerola pulp in different concentrations. It also evaluated the stability of beef (kebabs) and chicken (sashimi file) vacuum-packed with active films under refrigeration. The films and packaged products were characterized in relation to visual aspects, thickness, color parameters, pH, water vapor permeability, total phenolic content, pH, water holding capacity, shear strength, weight loss and biodegradability in soil essential for characterizing the films and verifying whether the packaging contributes to food preservation.

## 2. Materials and Methods

### 2.1. Materials

Commercial acerola pulp (Polpa Norte, Japurá, Paraná, Brazil), gelatin type A, Bloom 240, GAP 6 (Gelita^®^, Cotia, São Paulo, Brazil), and sorbitol (Dinâmica, Indaiatuba, Brazil) were used to produce active films. Beef cuts (counter fillet) and chicken (sassami fillet) were purchased in the local market in Dourados (Mato Grosso do Sul, Brazil). For the characterization and determination of the antioxidant capacity of the active films, sodium hydroxide (Dinâmica, Indaiatuba, Brazil), sodium chloride (Proquímicas, Salvador, Brazil), Folin–Ciocalteau (Dinâmica, Indaiatuba, Brazil), gallic acid (Dinâmica, Indaiatuba, Brazil), sodium carbonate (Impex, Sao Paulo, Brazil ), aluminum chloride (Synth), potassium chloride (Prochemicals, Rio de Janeiro, Brazil), sodium chloride (Prochemicals), sodium acetate (Prochemicals, Rio de Janeiro, Brazil), monopotassium phosphate (Vetec, Sao Paulo, Brazil), disodium phosphate (Vetec), hydrochloric acid (Vetec), Quercitin (Sigma, Sao Paulo, Brazil), ethyl alcohol (Neon, 95%, Rio de Janeiro, Brazil) and methyl alcohol (Chromate, Diadema, Brazil).

### 2.2. Characterization of Acerola Pulp

The moisture, ash content, and pH were determined according to Instituto Adolf Lutz [24]. The titratable acidity was determined according to the methodology described by Brasil [25]. Water activity and soluble solids content were determined using AquaLab (SERIESS 3TE) and refractometer (Atago, Master—80H, Tokio, Japan), respectively.

### 2.3. Active Film Production

The active films were produced using the casting technique. Different acerola pulp concentrations (60, 70, 80, and 90%) were incorporated, and 100% water was added to the mixture. The acerola pulp was thawed for 12 h in a refrigerator at 5 °C. After this period, the gelatin (5 g of gelatin per 100 g of filmogenic solution) was dispersed in the pulp and maintained at room temperature for 1 h for hydration. The sorbitol (20 g of sorbitol per 100 g of gelatin) was incorporated (under mechanical agitation, 3 min), and the filmogenic solution was maintained at 70 °C (thermostatic bath, Fisatom 5500, Sao Paulo, Brazil) under mechanical agitation (Fisatom 715, 500 rpm, 10 min, Sao Paulo, Brasil). The filmogenic solution was dispersed in polystyrene plates (12 × 12 cm) and dried at room temperature (25 ± 5 °C) for 24 h.

Before characterizations, the active films were stored in desiccators with NaBr saturated saline solution (58% relative humidity, 25 ± 5 °C).

### 2.4. Film Characterization

#### 2.4.1. Visual Aspects and Thickness

The visual aspects of the films were analyzed by homogeneity (uniform color and presence of insoluble particles). The thickness of the films (20 measurements taken randomly in the film area) was determined using a digital micrometer (0.001 mm, IP—65 model, Mitutoyo, Japan).

#### 2.4.2. Color Parameters and pH

The color parameters, brightness (L*), chroma a* (a*) e chroma b* (b*) of the films (4 × 4 cm) were determined using a Konica Minolta colorimeter (CR-400/410, illuminant D65, Tolio, Japan). The pH was determined according to the method proposed by Manhar and Suresh [26], in which the film samples (3 × 2 cm) were dispersed in 15 mL of saline phosphate-buffered solution (pH 6.8), prepared according to Föger et al. [27]. The pH was determined after 1 min using a pH meter (Bel, PHS3BW, Piracicaba, Brazil).

#### 2.4.3. Water Vapor Permeability (WVP)

Samples (5 cm in diameter) were fixed in the permeability cell. Cells were kept in desiccators containing a saturated saline solution of magnesium chloride (33% relative humidity) at 25 ± 2 °C (B.O.D Biothec incubator, BT 62). The weight gain was determined at 60 min intervals for 6 h. The water vapor permeability was calculated (Equation (1)) according to ASTM E 96 [28].

(1) $$WVP\;=\;\frac{G\;x}{t\;T_{a}\;W_{s}\;\left(R_{1}\;-\;R_{2}\right)}$$

where WVP = water vapor permeability (g.mm/h.m^2^.kPa); x = average film thickness (mm); $T_{a}$= transmission area (0.0015 m^2^); W_s_ = water saturation pressure (3.1690 kPa, 25 °C, and 100% RH); (R_1_ − R_2_) = relative humidity difference (100); G/t (g/h) = angular coefficient of the linear regression of the weight gain line as a function of time.

#### 2.4.4. Total Phenolic, DPPH and TEAC

The total phenolic content in the films was determined by the Folin–Ciocalteau method [29]. Film samples (0.05 g) were dispersed in 50 mL of distilled water and kept in an ultrasonic bath (Ultronique Q5.9/40 A, frequency 40 KHz and power 132 W) for 10 min. Subsequently, the dispersions were centrifuged at 3500 rpm for 5 min (ITR, Simplex II, Minas Gerais, Brazil). Aliquots of the supernatant (0.5 mL) were added to 2.5 mL of Folin–Ciocalteau (1:10), and after 5 min, 2 mL of sodium carbonate solution (7.5% in water) were added. The solution was homogenized (Phoenix-Luferco, AP 59, Araraquara, Brazil) and kept in the absence of light for 2 h. Absorbance was determined in a spectrophotometer (Marconi, Jenway 7310, Piracicaba, Brazil) at 740 nm, and the results expressed in mg equivalent of gallic acid (GAE) per 100 g of film.

The DPPH (2,2-diphenyl-1-picrylhydrazyl) radical scavenging activity was evaluated according to the method described by Brand-Williams et al. [30]. Films (0.2 g) were solubilized in distilled water using ultrasonic treatment to ensure complete dissolution. Aliquots of 2 mL of the solubilized films were mixed with 2 mL of a 0.2 mM DPPH• solution (A1). Control solutions included A2 (2 mL of film solution + 2 mL of distilled water) and A3 (2 mL of DPPH• solution + 2 mL of ethanol), as described by Li et al. [31]. After 3 h of reaction, absorbances (A1, A2, A3) were measured at 517 nm, and antioxidant capacity was calculated using Equation (2).

(2) $$Antioxidant\;capacity\;(\%)=\left[\left(1-\frac{A_{1}-A_{2}}{A_{3}}\right)100\right]$$

The antioxidant activity of the ferric-reducing antioxidant power (FRAP) method was determined following the procedure described by Benzie and Strain [32]. Samples of films (0.02 g) were dispersed in water in a 10 mL volumetric flask and subjected to ultrasonic treatment for 10 min to ensure complete solubilization. An aliquot of 150 μL of the solubilized film was mixed with 2850 μL of the FRAP reagent, and the mixture was incubated at 37 °C for 30 min. The absorbance was then measured at 593 nm. A calibration curve was prepared using Trolox as the standard, and the results are expressed as μM Trolox equivalents per gram of film.

### 2.5. Applications of Active Films in Meat Products

The active films with superior antioxidant activity were applied to meat products. Beef samples (BS) were cut with 2 cm thickness, and chicken samples (CS,) were used. Initially, three parts of the films were manually sealed using two films for each sample, then the meat products were added inside and sealed using a vacuum sealer (Panoran, Panvac 330, Espirito Santo, Brazil). The packaged samples (meat product + film) were refrigerated at 5 ± 2 °C for 9 days. The meat samples were analyzed at 1, 2, 3, 5, and 9 days. Additionally, beef and chicken (in natura) were stored under the same conditions without using the film (control).

#### 2.5.1. Color Parameters

The meat products were removed from the gelatin-based films with the incorporation of acerola pulp at intervals of 1, 2, 3, 5, and 9 days. The color parameters, lightness (L*), chroma a* (a*), and chroma b* (b*) were determined using a Konica Minolta colorimeter (CR-400/410, illuminant D65). The color parameters were measured in 10 random points of the BS and CS. The total color difference (∆E*) was calculated according to Equation (3), [33] using meat products as a default before adding them to the package.

(3) $${∆E}^{*}=\sqrt{{∆a}^{*2}+{∆b}^{*2}+{∆L}^{*2}}$$

#### 2.5.2. pH

The pH of the meat samples was determined on the first and ninth days of storage using a previously calibrated digital portable pH meter (Testo 205, Testo, Lenzkirch, Germany) that was introduced directly into the muscle portion of the meat products.

#### 2.5.3. Water Holding Capacity (WHC) and Shear Strength (SS)

The meat samples were cooked in an electric oven (Fischer Autolimpante Grill 44L—1323/5697, Madrid, Spain) preheated (170 °C) until they reached an internal temperature of 70 °C. The samples were removed from the oven and kept at room temperature until 28 °C. After cooking, the compression method determined the water holding capacity (WHC) in meat products [33]. A weight (2.25 kg) was maintained on the meat product samples (2 × 2 cm) for 5 min, and the final weight was measured. The WHC was calculated according to Equation (4).

(4) $$WHC\;=\;\left(\frac{W_{f}}{W_{i}}\right)\;\times \;100$$

where: WHC = water holding capacity (%); Wi = sample initial weight (g); Wf = final sample weight (g) after the weight is held over the sample.

To determine the shear strength, cylindrical samples (1.3 cm in diameter) were cut parallel to the orientation of the muscle fibers using a Warner-Bratzler Meat Shear Force instrument (Warner-Bratzler Meat Shear; G-R Manufacturing, Collins, KS, USA). The analysis was performed in a texturometer (TextureAnalyser, TA.XTplus, Cuidade de Mexico, Mexico). The pre-test, test, and post-test velocities were set at 10 mm/s, 2 mm/s, and 15 mm/s, respectively. A 1.0 mm thick Warner-Bratzler standard shear blade was used.

#### 2.5.4. Weight Loss (WL)

The weight loss of the meat samples was determined at 1, 2, 3, 5, and 9 days using an analytical balance (Ohaus, PA214CP, Barueri, Brazil) and determined according to Equation (5).

(5) $$WL\;(\%)\;=\;\left(\frac{P_{I}-P_{N}}{P_{I}}\right)\;\times \;100$$

where WL = percentage of sample weight loss (%); P_i_ = the initial weight of the sample on the first day of storage (g); P_n_ = sample weight for each evaluation day (g).

### 2.6. Biodegradability in Soil

**Figure 1:**
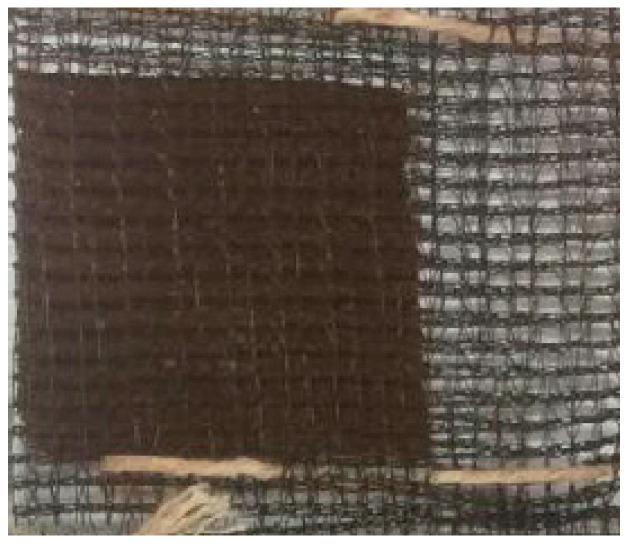
Bags produced from the plastic screen for storage with film samples for biodegradability in soil.

The biodegradability of the films was performed according to the methodology described by Maran et al. [34], with some modifications, using the sample with 90% (*w/w* of gelatin) of acerola pulp. Film samples (4 × 4 cm) were stored in bags (5 × 5 cm) produced from a plastic screen (50 mm), as shown in Figure 1. The samples were buried 8 cm deep in the soil (pH = 5.09, Experimental Farm of Agricultural Sciences—FAECA/UFGD). The samples were taken from the soil at regular intervals (5 days), and a visual analysis was carried out, observing the fissures or cracks.

### 2.7. Statistical Analysis

The analyses were performed in triplicate with 3 points in each repetition, totaling 9 samples in each analysis for the characterization tests of the acerola pulp and gelatin-based films, with and without the addition of acerola pulp. The analyses of meat product tests conditioned were performed in duplicate with 3 points in each repetition, totaling 6 samples in each analysis. Significant differences between results were evaluated using analysis of variance (ANOVA) and Tukey’s test, using the Statistica 8.0 software (STATSOFT TM, Inc., Tulsa, Oklahoma, USA) and a 95% confidence interval.

## 3. Results and Discussion

### 3.1. Characterization of Acerola Pulp

Frozen acerola pulp has 93.6% moisture and 0.3% ash (Table 1). The results obtained for moisture and ash are in accordance with the Brazilian Food Composition Table [35]. The value of solid soluble content was similar to that reported by Moura et al. [17] for acerola pulp (6.7 to 12.7 °Brix). There is a high variation in the content of soluble solids in acerola pulp due to the heterogeneity of the raw material, which is directly associated with genetic variability, degree of maturation, and environmental conditions [22]. The pH found for the acerola pulp was 3.7.

All the pulp characterization results were within the Identity and Quality Standards [34] for acerola pulp, which requires a minimum content of soluble solids of 5.5 °Brix and a titratable acidity of at least 0.80 g/100 g.

### 3.2. Film Characterization

#### 3.2.1. Visual Aspects, Thickness, and Color Parameters

The reddish color of active films was improved, increasing the concentration of acerola pulp, which is associated with a visual characteristic of the pulp (Figure 1). The formation of a continuous matrix was verified, with small heterogeneous zones (characteristics of pulps) and ease of support removal. There was a significant increase in the thickness of active films, increasing the concentration of acerola pulp, associated with the higher total solids (Table 2). Dantas et al. [36] reported the same behavior for cassava starch films, plasticized with glycerol and incorporated with acerola pulp, observing thickness ranging from 0.13 to 0.25 mm, containing 5 to 25% of pulp, respectively.

**Figure 2:**
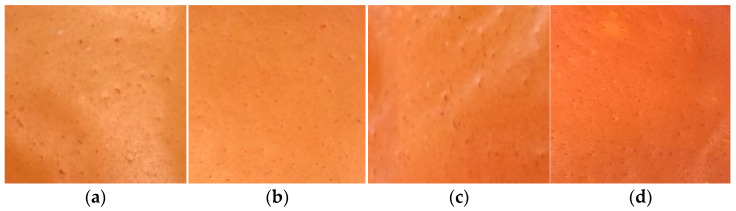
Gelatin-based films incorporated with different concentrations of acerola pulp, as follows: (**a**) 60 g per 100 g filmogenic solution, (**b**) 70 g per 100 g filmogenic solution, (**c**) 80 g per 100 g filmogenic solution, and (**d**) 90 g per 100 g filmogenic solution.

The values of the color parameters (Table 2) corroborate the visual aspect observed by red color intensity (Figure 2). With increasing concentration of incorporated pulp, L* values reduced, and a* and b* values increased. This is likely related to pigments in the acerola pulp that confer a reddish color.

The addition of acerola pomace extract to gelatin films changed the films’ color as a function of the extract concentration added. The L* and b* values were similar to those observed by Ribeiro et al. [37]. However, the a* value was higher, indicating a more intense red color.

#### 3.2.2. pH and Water Vapor Permeability

The pH of active films did not significantly change with 70, 80, and 90 g of acerola pulp/100 g of filmogenic solution (Table 2). However, the formulation with 60 g of acerola pulp/100 g of filmogenic solution differed significantly from the other formulations due to the lower concentration of added pulp.

The water vapor permeability of packages is a parameter that directly impacts the shelf life of packaged food products, estimating the water molecule diffusion rate within the film matrix. High values of water vapor permeability can accelerate quality deterioration and microbial spoilage reactions [38]. The water vapor permeability of active films with higher pulp concentrations (80 and 90 g of acerola pulp/100 g of filmogenic solution) was significantly higher than the other concentrations.

Changes in water vapor permeability values may be related to increased solids content in the polymeric matrix and concentrations of phenolic compounds in acerola pulp. According to Ramírez et al. [39], both should be considered relevant for water vapor permeability. They can interact with the polymer, making the matrix more compact, mainly due to the chemical interaction between the polymer and the active compounds. The presence of antioxidant compounds possibly reduced the hydrophobic groups in the gelatin matrix, leading to more significant interaction with water. In films based on blends of sago starch and fish gelatin plasticized with glycerol or sorbitol, gelatin is more hygroscopic than sago starch. Consequently, the affinity for water molecules will be greater in gelatin films, resulting in greater diffusion of water and films with higher WVP [40]. As gelatin already presents this high permeability to water molecules (highly hygroscopic), adding acerola pulp possibly resulted in greater diffusion of water through the films, influencing the water vapor permeability values.

#### 3.2.3. Total Phenolic Compounds, DPPH and FRAP

A significant increase in the concentration of phenolic compounds was verified by increasing the pulp concentration in the films (Table 2). The highest phenolic compound concentration was observed in the film with 90 g of acerola pulp/100 g of filmogenic solution. The composition of acerola processing by-products may contain higher levels of carotenoids, total anthocyanins, phenolic compounds, and flavonoids [20]. Studies in the literature have demonstrated the presence of phenolic compounds in commercial pulps [17,41].

The results obtained for the antioxidant activity of the gelatin films with different acerola pulp concentrations indicated that increasing the amount of pulp did not result in statistically significant differences, either in free radical scavenging activity (DPPH•) or in ferric-reducing antioxidant power (FRAP). For the DPPH assay, the values ranged from 84.36 ± 0.75% to 84.60 ± 0.85%, demonstrating a high antioxidant activity of the films regardless of the amount of pulp added. This stability suggests that, from 60 g of pulp, the bioactive compounds responsible for the antioxidant activity are already present at levels sufficient to saturate the film matrix.

The same trend was observed for the FRAP results. Furthermore, the behavior observed may be related to the chemical nature of the antioxidants present in acerola pulp, predominantly water-soluble compounds, which are easily incorporated into the gelatin matrix but whose overall antioxidant activity is not linearly increased with higher pulp concentrations. According to Laurindo et al. [42]., acerola is recognized for its exceptionally high content of vitamin C, carotenoids, and phenolic compounds, which contribute to its potential in reducing inflammation, oxidative stress, and the risk of metabolic diseases; both the pulp and processing residues can be effectively utilized in the development of juices, various food products, as well as in the production of antioxidants, natural additives, colorants, edible films, and prebiotics.

### 3.3. Evaluation of the Stability of Meat Products

Based on preliminary tests, the active film package selected to continue with the application in meat products was 90 g of acerola pulp/100 g of filmogenic solution, which showed a higher concentration of total phenolic compounds and stability in relation to the application of vacuum (Figure 2).

#### 3.3.1. Visual Aspects, pH, and Color Difference

After the 9-day storage period, the packages did not present a loss of vacuum. There was an increase in the pH value of the meat after 9 days of storage (Table 3). After slaughtering animals, the muscle pH is around 7.0. The lactic acid produced by the reserve glycogen is no longer transported to the liver to be metabolized during bleeding. It accumulates in the muscle tissue, reducing the pH to 5.8–5.5 24 h after slaughter [43]. There was an increase in pH values during storage (9 days), differing significantly in relation to 1st day for both cuts of meat (BS and CS), possibly due to the product’s interaction with the packaging.

The color difference (∆E*) showed a more significant variation in the CS compared to the BS (Table 3), differing significantly in the analyzed range. This was expected since the chicken meat has a lighter color, and films with acerola pulp showed a tendency to color red, justifying this color difference. Possibly, the color change is associated with the migration of pigments present in the incorporated pulp of films to the meat products.

The pH values increased on day 9, and ∆E* increased in meat products without films, differing significantly from beef and chicken with film (Table 3). This could be associated with the lack of protection of the meat products without the films. Possibly, the less significant increase in pH values occurred because the films acted as a barrier to external effects, in addition to interacting with the meat products leading to a smaller change in pH values. However, these values (~6.0) are close to the pH of the meat after the postmortem period and the accumulation of lactic acid.

#### 3.3.2. Water Holding Capacity and Shear Strength

The water holding capacity of meat products can affect the appearance of the meat before cooking. Its behavior during cooking and juiciness during mastication are important criteria for evaluating meat quality [44]. Meat products packaged with gelatin-based films incorporating acerola pulp had a water retention capacity of ~99% (Table 3). Moisture retention during cooking may have been helped by gelatin-based films incorporated with 90 g of acerola pulp/100 g of filmogenic solution, as a more significant loss of WHC was observed in meat products without adding film. The high water retention capacity means that weight loss is lower, indicating maintenance of the palatability and nutritional value of the meat [45]. The high water retention capacity observed in meat may be associated with maintaining pH values during storage. Reduced pH values may be associated with more intense glycolysis postmortem, increasing water loss through muscle protein denaturing [46].

**Figure 3:**
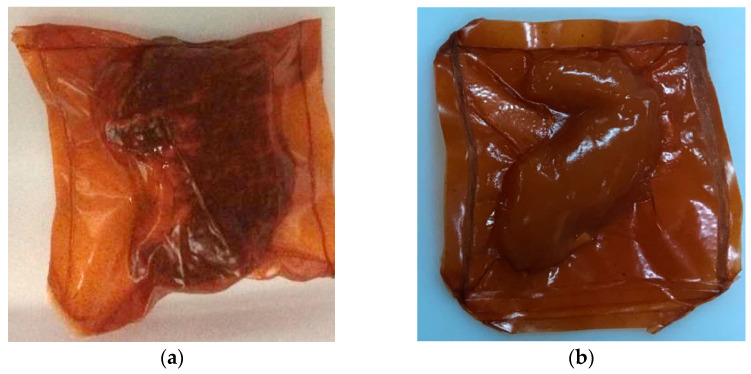
Vacuum-packed meat products with gelatin-based films incorporated with 90 g of acerola pulp per 100 g filmogenic solution: (**a**) Beef (BS) and (**b**) Chicken (CS).

Cooking meat significantly influences the texture due to protein denaturation, moisture loss, collagen, and muscle fiber shrinkage. Generally, these changes result in toughening the meat and increasing the shear values of cooked meat [47]. The texture of meat products was evaluated through the shear force after cooking (Table 3). The films contributed to maintaining the tenderness of the meat products since they presented values around 30% lower than the cuts without the application of films. Possibly, meat products without film application lose more moisture to the external environment, which can be proven by the mass loss data (Figure 3), resulting in meat hardening and greater shear force (Table 3).

According to Ramos and Gomide [48], the texture is extremely soft when presented with values lower than 3.6 kgf, slightly soft between 6.6 and 9.6 kgf, and slightly hard when above 12.60 kgf. Meat products vacuum-packed with gelatin-based films incorporating acerola pulp were considered soft, with values between extremely and slightly soft texture, possibly due to the observed water capacity increases (Table 3).

#### 3.3.3. Weight Loss

**Figure 4:**
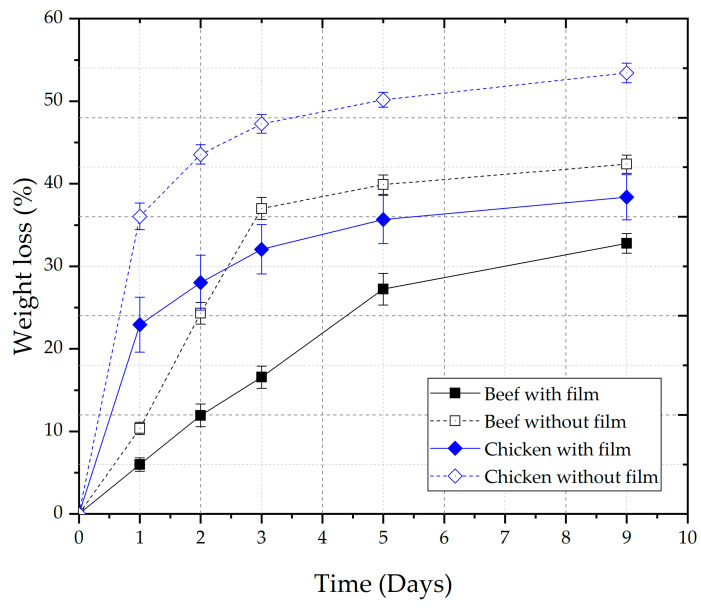
Weight loss of meat products (BS and CS) with and without packaging with gelatin-based films incorporated with 90 g of acerola pulp/100 g filmogenic solution.

Meat products without packaging were used as a control. After 9 days of storage under the same conditions, weight losses of ~42% and ~53% for beef and chicken without the films were found, respectively. The weight losses of approximately 33% and 39% for the sirloin fillet and the chicken were verified after 9 days of storage, indicating that the active films reduced the loss of mass in relation to unpackaged products, acting as a barrier (Figure 4).

### 3.4. Biodegradability in Soil

**Figure 5:**
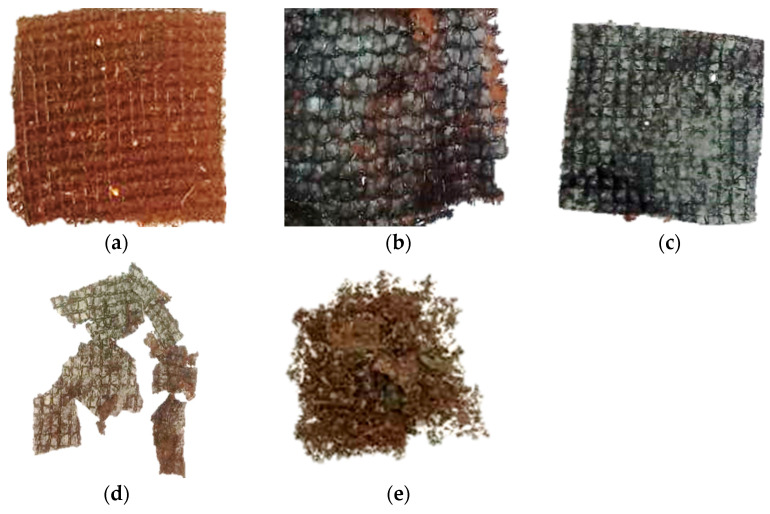
Biodegradability in the soil of the gelatin-based films incorporated with 90 g of acerola pulp/100 g filmogenic solution (without bags from the plastic screen): (**a**) 1 day, (**b**) 5 days, (**c**) 15 days, (**d**) 20 days, and (**e**) 25 days.

The films showed darkening after 5 days of contact with the soil. This is possibly due to microorganisms in the soil (Figure 5b). The films showed more significant macroscopic deterioration in the function of the films during the experiment, in which the films lost their initial appearance and structural integrity, clearly demonstrating their degradability (Figure 5c,d). After 25 days, it was not possible to recover the entire film from the soil, and it was possible to confirm its biodegradation in the soil. During the experiments, a wide variation in humidity and temperature was observed due to the characteristics of the region’s climate, which possibly favored the biodegradation process of the films.

The biodegradation of films involves the medium where they are stored, and climatic conditions, which are physical or chemical modifications, are influenced mainly by heat and humidity [49]. Abrusci et al. [50] reported that soil microflora is a mixed microbial population reproducing in naturally occurring conditions. In addition, microorganisms produce enzymes that react with surface proteins.

## 4. Conclusions

Acerola pulp incorporated in gelatin-based films could be an alternative to replace or minimize the use of non-biodegradable packaging, reducing the environmental impacts. Additionally, it could be applied as active packaging due to the antioxidant properties observed by incorporating acerola pulp. After application of the films (90 g of acerola pulp/100 g of filmogenic solution) on beef and chicken, the samples showed greater water retention capacity, with reduced shear force values, which contributed to the longer shelf life of meat products, making it possible to use biodegradable packaging in meat products. After 20 days, it was not possible to separate the film from the soil, confirming its biodegradability.

## Figures and Tables

**Table 1:** Characterization of acerola pulp for incorporation in active films.

Property	Results
Moisture (%)	92.93 ± 0.06
Ashes (%)	0.48 ± 0.04
pH	3.74 ± 0.04
Titratable acidity (g/100 g citric acid)	13.77 ± 0.33
Water activity (Aw)	0.98 ± 0.01
Total soluble solids (°Brix)	7.37 ± 0.16

**Table 2:** Thickness (mm), surface pH, water vapor permeability (WVP, g mm/h m^2^ kPa), color parameters (luminosity—L*; chroma a*—a* and chroma b*—b*), and total phenolic compounds (TFC, mg GAE/100 g) of gelatin-based films incorporated with different concentrations of acerola pulp (60, 70, 80, and 90 g per 100 g filmogenic solution).

Analysis	Concentrations of Acerola Pulp (g/100 g Filmogenic Solution)			
	60	70	80	90
Thickness	0.10 ± 0.01 ^c^	0.11 ± 0.01 ^bc^	0.13 ± 0.02 ^ab^	0.14 ± 0.02 ^a^
pH	6.30 ± 0.19 ^a^	5.98 ± 0.08 ^b^	5.93 ± 0.21 ^b^	6.06 ± 0.20 ^b^
WVP	1.48 ± 0.04 ^c^	1.43 ± 0.03 ^c^	1.71 ± 0.04 ^b^	1.90 ± 0.06 ^a^
Color parameters				
L*	76.61 ± 0.82 ^a^	73.45 ± 1.09 ^b^	69.75 ± 1.42 ^c^	67.55 ± 1.23 ^d^
a*	20.34 ± 1.03 ^d^	24.57 ± 1.26 ^c^	29.20 ± 1.62 ^b^	32.85 ± 2.41 ^a^
b*	40.84 ± 3.85 ^c^	45.97 ± 1.87 ^b^	51.09 ± 1.70 ^a^	53.43 ± 1.66 ^b^
TFC	2884.17 ± 70.24 ^d^	3108.17 ± 72.46 ^c^	3609.51 ± 85.68 ^b^	3935.02 ± 96.05 ^a^
DPPH ^1^	84.36 ± 0.75 ^a^	84.45 ± 0.80 ^a^	84.36 ± 0.75 ^a^	84.60 ± 0.85 ^a^
FRAP	1.05 ± 0.09 ^a^	1.07 ± 0.04 ^a^	1.08 ± 0.09 ^a^	1.10 ± 0.12 ^a^

Lowercase letters in the same lines indicate significant differences (*p* < 0.05). ^1^ DPPH scavenging activity.

**Table 3:** pH, the color difference (∆E*), water holding capacity (WHC), and shear strength (SS) of meat products (beef and chicken) with and without packaged with films with the incorporation of acerola pulp (90 g of acerola pulp/100 g filmogenic solution).

Analysis	BS	BS Without Film	CS	CS Without Film
pH_Day1_	-	5.68 ± 0.03	-	5.84 ± 0.03
pH_Day9_	6.00 ± 0.04 ^bA^	6.28 ± 0.16 ^aB^	5.95 ± 0.01 ^bA^	6.51 ± 0.02 ^aA^
∆E*	7.88 ± 0.62 ^bA^	13.08 ± 1.0 ^aB^	18.05 ± 0.17 ^bB^	21.80 ± 1.90 ^aA^
WHC (%)	98.70 ± 0.15 ^aA^	98.65 ± 0.16 ^aA^	98.14 ± 1.13 ^aA^	90.74 ± 12.88 ^bB^
SS (kgf)	2.45 ± 0.17 ^bB^	3.46 ± 0.59 ^aB^	3.91 ± 2.08 ^bA^	5.81 ± 0.24 ^aA^

Lowercase letters in the same column indicate significant differences (*p* < 0.05) for the same analysis between the meat product (BS and CS) with and without film addition. Capital letters in the same column indicate significant differences (*p* < 0.05) between beef and chicken and between beef without film and chicken without film.

## Data Availability

The data is contained in the article.
